# Management of stage II colon cancer - the use of molecular biomarkers for adjuvant therapy decision

**DOI:** 10.1186/1471-230X-13-36

**Published:** 2013-02-27

**Authors:** Marisa Donada, Serena Bonin, Renzo Barbazza, Daniel Pettirosso, Giorgio Stanta

**Affiliations:** 1 DSM Department (Department of medical, surgical and health sciences), University of Trieste, Surgical Pathology Bldg, Strada di Fiume 447, I-34149, Trieste, Italy

**Keywords:** Colon cancer, Stage II, Adjuvant therapy, 5-Fluorouracil, Formalin-fixed and paraffin-embedded tissues, Thymidylate synthase, MMR, CIMP

## Abstract

**Background:**

There is uncertainty on the benefit of adjuvant chemotherapy in patients with stage II colorectal cancers. The aim of this study is to investigate the combined role of clinical, pathological and molecular parameters to identify those stage II patients who better benefit from adjuvant therapy.

**Methods:**

We examined 120 stage II colon cancer patients. Of these, 60 patients received adjuvant 5-FU chemotherapy after surgery and the other 60 did not receive therapy. Immunohistochemical (IHC) analyses were performed to evaluate the expressions of Thymidylate synthetase (TYMS), TP53 (p53), β-catenin (CTNNB1) and CD8. For TYMS, its mRNA expression levels were also investigated by real time qRT-PCR. The entire case study was characterized by the presence of a defect in the MMR (mismatch repair) system, the presence of the CpG island methylator phenotype (CIMP or CIMP-High) and for the V600E mutation in the BRAF gene. At the histo-pathological level, the depth of tumour invasion, lymphovascular invasion, invasion of large veins, host lymphocytic response and tumour border configuration were recorded.

**Results:**

The presence of the V600E mutation in the BRAF gene was a poor prognostic factor for disease free and overall survival (DFS; hazard ratio [HR], 2.57; 95% CI: 1.03 -6.37; p = 0.04 and OS; HR, 3.68; 95% CI: 1.43-9.47; p < 0.01 respectively), independently of 5-FU treatment. Adjuvant therapy significantly improved survival in patients with high TYMS levels (p = 0.04), while patients with low TYMS had a better outcome if treated by surgery alone (DFS; HR, 6.07; 95% CI, 0.82 to 44.89; p = 0.04). In patients with a defect in the MMR system (dMMR), 5-FU therapy was associated to reduced survival (DFS; HR, 37.98; 95% CI, 1.04 to 1381.31; p = 0.04), while it was beneficial for CIMP-High associated tumours (DFS; HR, 0.17; 95% CI, 0.02 to 1.13; p = 0.05).

**Conclusions:**

Patients’ characterization according to MMR status, CIMP phenotype and TYMS mRNA expression may provide a more tailored approach for adjuvant therapy in stage II colon cancer.

## Background

Colorectal cancer (CRC) is the third most common tumour in men and the second in women worldwide and it is the fourth most common cause of death from cancer [1].

Colorectal cancer has usually been viewed as a homogeneous entity rather than a complex heterogeneous disease developing through multiple genetic and epigenetic abnormalities, such as defective DNA mismatch repair (dMMR) and the CpG island methylator phenotype (CIMP) [2,3]. The functional consequence of failure of the MMR system is a high-frequency of microsatellite instability (MSI-H), the condition in which cancers show an increased rate of replication errors in the highly repetitive short DNA sequences called microsatellites [4]. CpG island methylator phenotype (CIMP or CIMP-High) comprises a subset of colorectal carcinoma characterized by extensive hypermethylation of multiple promoter CpG island loci and peculiar clinico-pathologic features [3]. In addition to CIMP-High, recent findings have identified the CIMP-Low cancer subtype, which appears to be a distinct biological subtype of colorectal cancer with respect to CIMP-High and which is characterized by less extensive CIMP-specific promoter methylation [5,6].

Adjuvant chemotherapy, mostly based on the use of the antimetabolite 5-Fluorouracil (5-FU), is mainly provided for patients with stage III CRCs, whereas evidence on the usefulness of adjuvant therapy in stage II CRC patients is controversial [7]. Current treatment protocols recommend adjuvant treatment only to stage II patients with high-risk pathological features (e.g. T4 stage, bowel perforation or clinical bowel obstruction, inadequate lymph node sampling, poorly differentiated histology) [8,9]. As to histo-pathological characteristics, other high-risk features such as lympho-vascular invasion [10,11], absence of tumour inflammatory response [12,13] and presence of tumour budding [14] are also suggested as indicating a higher risk of recurrence and hence a worse prognosis.

The histo-pathological approach is paramount in cancer classification, however for most patients with stage II disease who are classified as standard risk, there are no additional markers to refine risk assessment or to predict adjuvant chemotherapy benefit. This limitation could be overcome by the use of molecular biomarkers in addition to pathological classification, aiming at a more personalized approach in cancer treatment. Molecular markers may have prognostic or predictive value. Prognostic biomarkers provide information on the clinical outcome at the time of diagnosis, independently of therapy, whereas predictive biomarkers provide information about the likelihood of response to a given therapeutic modality based on marker status, and therefore could be used to guide treatment [15]. In colorectal cancer, several potential molecular predictors of recurrence risk and chemotherapy benefit have been investigated [16]. Previous reports suggested that patients with a defective DNA mismatch repair system (dMMR) show improved prognosis over those with proficient mismatch repair (pMMR) [17] and they do not receive benefit from 5-FU based adjuvant therapy [18,19]. Moreover, recent findings highlighted that benefit to 5-FU based adjuvant therapy in dMMR patients was restricted to suspected germline (ie Lynch syndrome) vs sporadic tumours, especially in stage III cancers [20].

Unlike MMR studies, reports focusing on the relationship between CIMP status and survival have yielded contradictory results. Some authors have suggested that patients with CIMP colorectal tumours do not benefit from 5-FU-based adjuvant chemotherapy [21], while others have observed an independent association of CIMP status to lower specific cancer mortality [22]. Many other single molecular markers have been investigated as prognostic or predictive in CRC [23]. Among them, the most studied is Thymidylate synthetase (TYMS), which has been extensively studied as a predictive factor for response to adjuvant fluoropyrimidine chemotherapy. TYMS is the key enzyme for the cellular production of the thymidine monophosphate (dTMP), which is considered the main target of the 5-FU. Some studies reported a positive relationship between TYMS expression and survival [24], while others showed a negative relationship [25] or no correlation [26,27].

The identification of prognostic and predictive markers of therapy efficacy for stage II CRC is pressing because the rate of recurrence for patients with stage II colon carcinoma was reported to be around 15% [28] and stage II cancers represent 30-40% of all resected colorectal cancers. This percentage is going to increase because of the screening programmes [29]. This study aims to deepen investigation on the possibility of combining classical histo-pathological features with molecular alterations (at the genomic, transcriptomic and epigenomic level) for a more tailored adjuvant therapy in stage II colorectal cancer. For our study we selected as candidate molecular markers the colorectal cancer classifiers MMR system and CIMP, as well as the following single molecular markers: TP53 (p53), β-catenin (CTNNB1), BRAF c.1799 T > A (V600E) mutation and TYMS. The first three markers were chosen because they are involved in CRC tumorigenesis [30], whereas TYMS was analysed because it could be used to identify a subgroup of stage II colon cancer patients who better benefit from the use of adjuvant chemotherapy, as we pointed out in a previous study [31].

Among the pathological factors we considered the following ones, which have been recognized as prognostic in the assessment of the risk of recurrence in CRCs: the depth of tumour invasion [32], the lympho-vascular invasion and the invasion of large veins [10,11], the presence of a tumour inflammatory response [12,13] and the tumour border configuration [14].

## Methods

To identify prognosis-related and predictive markers of therapy efficacy in CRC, a cohort of 120 patients diagnosed with exclusively colon cancer of stage II and treated with surgery alone or surgery plus 5-FU based adjuvant chemotherapy were included in our survey. All molecular analyses were performed on formalin-fixed paraffin-embedded tissues (FFPE) specimens to directly compare experimental results with the patients’ clinical data and follow up.

### Patients

FFPE cancer tissues were obtained from 120 patients whose diagnosis was primary colon adenocarcinoma of stage II. These patients were treated with surgery alone (60 patients) or surgery plus 5-FU/LV (60 patients; 5-fluorouracil 370–420 mg/m^2^ plus leucovorin 20–200 mg/m^2^ on day 1–5, repeated every 4–5 weeks for 6 months). Patients were selected among over 1000 patients, diagnosed with colorectal cancer and treated at the Trieste University Hospital. Of these, only those patients with exclusively colonic tumours of stage II and for whom clinical follow-up and archival material was available were included in the study. Another criterion in selection was homogeneity in therapeutic treatment. In particular, none of the patients received any anticancer treatment prior to surgery and at the time of the first diagnosis they did not present with any other cancers. Patients receiving 5-FU therapy after surgery and those treated with surgery alone were matched according to sex and age. From each patient, normal distal colon tissue was also recovered. Patients were followed through the population-based Friuli–Venezia Giulia Cancer Registry from diagnosis of cancer to the appearance of the first recurrence and to death or until 15 December 2010, whichever came first. The study was submitted for evaluation to the Ethical Committee of the University of Trieste, who approved it.

### Pathologic examination

Pathologic examinations (on hematoxylin-eosin slides) were performed independently by two pathologists (G.S and R.B.) on the entire case study to confirm tumour grade and histotype components and to evaluate the following factors: depth of tumour invasion, lympho-vascular invasion, large veins invasion, tumour infiltrating lymphocytes, Chron’s like reaction and the configuration of the tumour border. Evaluations of such parameters were quoted as in reference literature [33]. In particular, in the evaluation of tumour infiltrating lymphocytes, tumours were considered infiltrated if five or more intra-tumour lymphocytes per high-power field were detected (92% of concordance of identification, Kappa value of 0.79). Lympho-vascular invasion, large veins invasion and Chron’s like reaction were dichotomized as “no” or “yes” according to their absence or evident presence (85%, 87% and 88% of identification concordance for the two pathologists for the three parameters, with corresponding kappa values of 0.64, 0.67 and 0.71 respectively). Finally, the tumour border configuration was defined as “pushing” when tumours were smooth and rounded and they appeared to push into adjacent tissues, while it was defined as “infiltrative” when an irregular leading edge of cells infiltrated normal tissues with small groups of separated cells (96% of concordance; kappa value = 0.89). Discrepancies in evaluations were solved by simultaneous re-examination of the slides by both investigators using a double-headed microscope.

### DNA isolation and CpG island methylation status analysis

DNA was extracted from FFPE tissues of each patient’s tumour after mechanical microdissection, as described elsewhere [34]. After DNA extraction, sodium bisulfite conversion of the DNA was performed using a homemade method as previously described [34]. A tumour was defined CIMP-High if it showed methylation in at least three of the five methylation markers (CACNA1G, IGF2, NEUROG1, RUNX3, and SOCS1), suggested by Weisenberger et al. [35]. CIMP-Low tumours were defined as tumours with 1/5 and 2/5 methylated promoters and CIMP-0 were those cancers with no methylated promoters. DNA methylation in the CpG islands of these five CIMP markers was determined by Methylation Specific PCR (MSP) as already reported [36,37]. Detection of the MSP reactions was done by non-denaturing poly-acrylamide electrophoresis, stained with ethidium bromide. Two assays were performed for each locus and a CpG-island was defined as methylated when both assays showed amplification. When one of two assays showed no amplification, a third assay was performed to ascertain whether that locus was methylated or not.

### BRAF c.1799 T > A mutation analysis (V600E)

BRAF c.1799 T > A mutation was searched by semi-nested PCR using the following primers: Forward outer primer: 5^′^-CTTGCTCTGATAGGAAAATGAGA-3^′^; Forward inner primer: 5^′^TGTTTTCCTTTACTTACTACAC-3^′^; Reverse primer: 5^′^-TCTTACCATCCACAAAATGGA-3^′^.

Amplicons span exon 15, codon 600, with a final inner amplicon length of 174 bp. PCR was performed under the following conditions: initial denaturation step of 95°C for 3^′^; 40 cycles of 95°C for 30 s; annealing at 51°C for 30 s; 72°C for 30 s; and a final elongation step of 72°C for 5^′^. One microliter of the first PCR reaction product was used as a template in the second PCR round. Thermal profile of the latter was the same as the first, despite the final number of cycles (30 cycles). The inner PCR products were first analyzed by 2.5% agarose gel electrophoresis, then purified using the QIAquick PCR purification kit (Qiagen) and submitted to direct sequencing. Standard dideoxy sequencing reaction and sequencing run were performed at the BMR-genomics sequencing core facility (http://www.bmr-genomics.it/; Padua, Italy).

### RNA isolation and TYMS analysis

For each patient, total RNA was extracted from FFPE specimens of primary colon adenocarcinomas after mechanical microdissection, as described elsewhere [34] and reverse-transcribed into cDNA after DNase treatment as previously reported [38]. TYMS mRNA expression levels were analysed by means of real time qPCR. Amplification was performed using a Mastercycler® ep realplex (Eppendorf, Hamburg, Germany) and data normalized as already reported [31].

### Tissue microarray (TMA) construction and immunohistochemistry

TMA blocks were prepared with inclusion of tumour and matched normal tissues, chosen by a pathologist. Areas without haemorrhage, necrosis, poor fixation or tissue processing artefacts were selected and punched with 1.5 mm sized cores. For specimens histologically non-homogeneous we extracted more tissue cores, representative of the different areas. For those cases an average of the count was used for the IHC analysis. The TMA blocks were cut in 4 μm sections for immunohistochemical staining. Immunostaining for each antigen was conducted using the avidin-biotin peroxidase complex technique (Vectastain Universal Elite ABC Kit, Vector Laboratories), following the manufacturer’s instructions. The antibodies TYMS (clone TS106, Millipore; diluted 1:50), MLH1 (clone G168-15, BD Pharmingen; diluted 1:25), MSH2 (clone G219-1129, BD Pharmingen; diluted 1:100), TP53 (clone DO-7, Ventana Medical Systems; pre-diluted), β-catenin (CTNNB1) (clone 14, Cell Marque; pre-diluted) and CD8 (clone SP57, Ventana Medical System; pre-diluted) were used. The IHC results were evaluated by two pathologists independently. In detail, for TYMS tumours were classified as “low” or “high” expression groups as already reported [26]. Tumours showing loss of nuclear MLH1 or MSH2 expression were classified as dMMR (i.e. with a defective MMR system), whereas the other tumours were defined as pMMR (i.e. with a proficient MMR system) as already suggested [19]. In 29 patients from the case study, MSI determination by microsatellite analysis [39] was also performed to check the equivalence of this method with the determination of the MMR system status through the immunohistochemical evaluation of MLH1 and MSH2.

TP53 immunoreactivity was dichotomised into positive and negative, based on staining of malignant nuclei, with a threshold of 10% [26]. For CTNNB1 (ß-catenin) evaluation, cytoplasmic, nuclear and membrane expressions were separately recorded as no or weak expression, moderate expression, or strong expression. Positivity in each compartment (cytoplasm, nucleus and membrane) was then defined as moderate/strong expression in that compartment as already described [40]. We also calculated the grades of activation of CTNNB1, as previously reported [40]. Immunoreactivity for CD8 was done by assessing the percentage of immunoreactive leukocytes in the tumoral area in a high magnification field, over the total number of leukocytes identified in the same field. The observations coming from three magnification fields were averaged.

### Statistical analysis

Associations between clinical-pathological data and categories of markers were tested for significance using the chi-square test (or Fisher’s exact test depending on the number of samples) for categorical variables. For continuous variables the parametric Student’s t-test or the one-way ANOVA test was used. Differences in the clinical, pathological and molecular characteristics between the group of patients treated with surgery alone and those submitted to adjuvant chemotherapy were evaluated with statistical tests for paired variables. The level of agreement between estimators was assessed using the Cohen’s kappa statistics. Real time qRT-PCR normalized values for TYMS were dichotomized for subsequent survival analysis with respect to the median value of expression. Tumours with gene expression levels lower or higher than the median value were classified as low or high status of expression, respectively. The primary end-point of the study was disease free survival (DFS), defined as the time from initial diagnosis to the first recurrence diagnosis. Overall survival (OS), which was defined as the time from surgery to colon cancer specific death, was the secondary end-point. In detail, in this study disease free patients were: 1) those who were alive and without recurrences at the end of the follow-up (15/12/2010); 2) those who died of causes not related to colon cancer and without recurrences during the follow-up (end of follow-up is the death date); 3) those who were alive and without recurrences, but emigrated (end of follow-up is the emigration date). To estimate the joint effects of the analysed covariates on patients’ survival, the data were analysed by fitting the Cox proportional hazard regression model. Cox proportional hazard analysis included: age at diagnosis, tumour location, tumour grade and the complete set of the pathological and molecular markers analysed. The log-rank test was used to check the dependence of patients’ survival on single variables or on combinations of variables. All p-values are two-sided with values <0.05 regarded as statistically significant. Although not really significant, P-values between 0.05 and 0.07 were considered “borderline”, as already done by other authors [41] since they could give some indication on the trend.

Statistical analyses were performed with the Stata/SE 9.2 package (Stata, College Station, TX).

## Results

### Description of patients’ cohort according to clinical, pathological and molecular features

The study population comprised 120 patients with stage II colon cancer who were divided into two subgroups according to the treatment after surgery. Patients belonging to the two subgroups were matched for sex and age. The median duration of overall follow up was 9.4 years (25th-75th percentile = 3.3-11.8 yrs). Clinical-pathological and molecular reports of the patients are reported in detail in Table 1. The features of patients treated with surgery alone and of those treated with surgery plus 5-FU adjuvant therapy are similar.

**Table 1:** Clinical, pathological and molecular characteristics of colon cancer patients in the two cohorts of treatment

			**Frequency N (%)**		
			**Surgery alone (n = 60; 50%)**	**5-FU therapy (n = 60; 50%)**	**p**
**Clinical-pathological features**	**Age, mean (SD), years**		67.4 (10.44)	67.7 (10.8)	0.9
	**Sex**	Male	28 (47)	29 (48)	0.3
		Female	32 (53)	31 (52)	
	**Tumour location**^**1**^	Proximal	21 (35)	25 (42)	0.4
		Distal	39 (65)	35 (58)	
	**Tumour grade**	G1	8 (13)	8 (13)	0.5
		G2	51 (85)	49 (82)	
		G3	1 (2)	3 (5)	
	**Depth of tumour invasion**	pT3	53 (88)	53 (88)	1.0
		pT4	7 (12)	7 (12)	
	**Lymphovascular invasion**	No	39 (65)	37 (62)	0.8
		Yes	21 (35)	22 (38)	
	**Large veins invasion**	No	51 (85)	47 (78)	0.3
		Yes	9 (15)	13 (22)	
	**Tumour infiltrating lymphocytes**	No	53 (88)	48 (80)	0.2
		Yes	7 (12)	12 (20)	
	**Chron’s like reaction**	No	31 (52)	27 (45)	0.4
		Yes	29 (48)	33 (55)	
	**Tumour border configuration**	Pushing	56 (93)	51 (85)	0.2
		Infiltrating	4 (7)	9 (15)	
**Molecular parameters**	**MMR system**	dMMR	8 (13)	10 (17)	0.6
		pMMR	52 (87)	50 (83)	
	**CIMP**	CIMP-High	8 (13)	14 (23)	0.3
		CIMP-Low	18 (30)	18 (30)	
		CIMP-0	34 (57)	28 (47)	
	**TYMS mRNA expression**	High level	24 (40)	33 (55)	0.1
		Low level	36 (60)	27 (45)	
	**TYMS protein expression (IHC)**	High	30 (50)	28 (47)	0.7
		Low	30 (50)	32 (53)	
	**TP53**	Positive	24 (40)	22 (37)	0.7
		Negative	36 (60)	38 (63)	
	CTNNB1^**2 **^**Cytoplasmic localization**	Positive	41 (68)	41 (69)	0.8
		Negative	19 (32)	18 (31)	
	CTNNB1^**2 **^**Nuclear localization**	Positive	18 (30)	26 (44)	0.1
		Negative	42 (70)	33 (56)	
	CTNNB1^**2 **^**Membrane localization**	Positive	27 (45)	19 (32)	0.1
		Negative	33 (55)	40 (68)	
	**BRAF c.1799 T > A**	Wild type	51 (85)	52 (87)	1.0
		Mutated	9 (15)	8 (13)	

^1^: Proximal colon includes cecum to transverse colon and distal colon includes splenic flexure to sigmoid colon.

^***2***^: One of the 60 cases treated with 5-FU was excluded from the analysis because of poor staining.

Immunohistochemical analysis showed that the percentage of intra-tumoral cytotoxic T lymphocytes (CD8+) was higher in cases classified as having tumour infiltrating lymphocyes and indicated that these infiltrating lymphocyes were mostly cytotoxic T lymphocytes (CD8+) (p < 0.01). The presence of tumour infiltrating lymphocytes was associated with the presence of a Chron’s like reaction in the border of the tumour (p < 0.01). For this reason these two features are analyzed together and referred to as “tumour inflammatory response” hereinafter.

### Molecular marker analysis and relationship with clinical and pathological features

Fifteen per cent of tumours were classified as dMMR. Deficient MMR tumours developed at higher frequencies at proximal sites than pMMR ones (p < 0.01) and tended to be less differentiated (p < 0.01). The presence of a tumour inflammatory response was also more frequent in patients with dMMR tumours (p = 0.03) (Table 2). Eighteen per cent of tumours were classified as CIMP-High, 30% were defined as CIMP-Low, while CIMP-0 comprised 52% of all tumours. CIMP-High and CIMP-0 tumours were more common in women, while CIMP-low were more common in men (p = 0.02; Table 2). CIMP-High phenotype was significantly associated with poor histological grade (p = 0.04) and dMMR (p < 0.01). In detail, 50% of CIMP-High tumours showed a defect in the MMR system, compared with only 5% of CIMP-Low and 8% of CIMP-0 ones. Moreover, tumour location bowel sub-site (from cecum to descending colon) was significantly linearly associated with dMMR (p < 0.01), but not with CIMP-High (p < 0.2). In detail, the frequencies of dMMR tumours were significantly decreasing from cecum to sigma.

**Table 2:** **Clinical and molecular characteristics of colon cancer patients according to MMR and CIMP**^**1**^

			**Molecular classifiers**			
			**MMR system frequency N%**		**CIMP status frequency N%**	
**Clinical and pathological parameters**			**pMMR dMMR**	**p**	**CIMP-0 CIMP-L CIMP+**	**p**
			**102 (85) 18 (15)**		**62 (52) 36 (30) 22 (18)**	
	**Sex**	Male	50 (59) 7 (39)	0.4	24 (39) 24 (67) 9 (41)	**0.02**
		Female	52 (41) 11(61)		38 (61) 12 (33) 13 (59)	
	**Tumour location**	Proximal	34 (33) 12 (67)	**<0.01**	25 (40) 9 (25) 12 (54)	0.07
		Distal	68 (67) 6 (33)		37 (60) 27 (75) 10 (46)	
	**Tumour grade**	G1	13 (13) 3 (17)	**<0.01**	8 (13) 6 (17) 2 (9)	**0.04**
		G2	88 (86) 12 (67)		54 (87) 29 (81) 17 (77)	
		G3	1 (1) 3 (17)		0 (0) 1 (2) 3 (14)	
	**Tumour inflammatory response**	No	51 (50) 4 (22)	**0.03**	26 (42) 20 (55) 9 (41)	0.4
		Yes	51 (50) 14 (78)		36 (58) 16 (45) 13 (59)	
**Molecular parameters**	**TS by mRNA**	High	44 (43) 13 (72)	**0.02**	22 (35) 19 (53) 16 (73)	**<0.01**
		Low	58 (57) 5 (28)		40 (65) 17 (47) 6 (27)	
	**TS by IHC**	High	52 (51) 6 (33)	0.2	30 (48) 16 (44) 12 (55)	0.2
		Low	50 (49) 12 (67)		32 (52) 20 (56) 10 (45)	
	**TP53**	Positive	44 (43) 2 (11)	**<0.01**	27 (44) 9 (25) 10 (45)	0.1
					35 (56) 27 (75) 12 (55)	
		Negative	58 (57) 16 (89)			
	***CTNNB1 *activation grades**	1	34 (33) 11 (66)	**0.02**	19 (31) 19 (53) 7 (32)	0.08
		2	54 (53) 3 (17)		33 (54) 15 (42) 9 (41)	
		3	14 (14) 3 (17)		9 (15) 2 (5) 6 (27)	
	**BRAF c.1799 T>A**	Wild	91 (89) 13 (72)	**0.05**	54 (87) 32 (89) 18 (82)	0.7
		Mutated	11 (11) 5 (28)		8 (13) 4 (11) 4 (18)	

^1^Significant or borderline p values are bolded.

Thymidylate synthetase expression was analysed both at the mRNA and at the protein level (by IHC). No correlation was observed between TYMS expression and any clinical-pathological variable, with the exception of tumour location, with higher TYMS mRNA expression in proximal tumours (p = 0.02). Interestingly, no correlation was found between TYMS mRNA expression levels and protein levels detected by IHC (p = 0.6). As to CTNNB1expression, one of the 120 cases was excluded from the analysis because of poor staining quality. Overall, 69% of tumours showed cytoplasmic positivity, 37% exhibited nuclear positivity and 39% displayed membrane positivity. Different types of positivity could be observed in the same sample. CTNNB1grades of activation were calculated as previously reported [40]. A strong relationship was found between CTNNB1activation degrees and CTNNB1expression according to localization, with nuclear/cytoplasmic expressions being more associated with higher degrees (p < 0.01). For this reason, to reduce the number of variables, in place of cellular localizations, the degrees of activation of CTNNB1 are considered in the analysis hereinafter.

TP53 showed nuclear immunoreactivity in 38% of the cases and BRAF c.1799 T > A mutation was detected in 13% of tumours. All these molecular parameters were unrelated with the clinical and pathological characteristics that were considered.

### Molecular correlates

Complex cellular events in CRC include CIMP and MSI. In our study we used immunohistochemistry of MLH1 and MSH2 as a surrogate marker of MSI, after demonstrating that immunohistochemical assessment of MLH1 or MSH2 corresponds to MSI testing. Concordance for the identification of a defect in the MMR system between IHC and MSI assessment by PCR was of 93% (Kappa value = 0.76; p < 0.01). Molecular correlations with CIMP and MMR are reported in Table 2. Thymidylate synthetase mRNA expression levels were significantly associated with the MMR system status and the CIMP status. In detail, 72% of dMMR patients expressed higher levels of the TYMS gene (p = 0.02) and 73% of patients with a CIMP-High status had a high TYMS status (p < 0.01) (Table 2). Conversely TYMS protein expression levels (by IHC) were found to be unrelated to MMR and CIMP (Table 2). TP53 positive staining was related to MMR system status: tumours lacking TP53 nuclear positivity were mostly dMMR, while nuclear positivity for p53 was related to pMMR (p < 0.01). No relationship was found between TP53 nuclear immunostaining and CIMP status (p = 0.1) (Table 2). Tumours with a dMMR were characterized by lower degrees of activation of CTNNB1 (p = 0.02) and the presence of c.1799 T > A mutation in the BRAF gene (p = 0.05) (Table 2).

### Survival analysis

**Figure 1:**
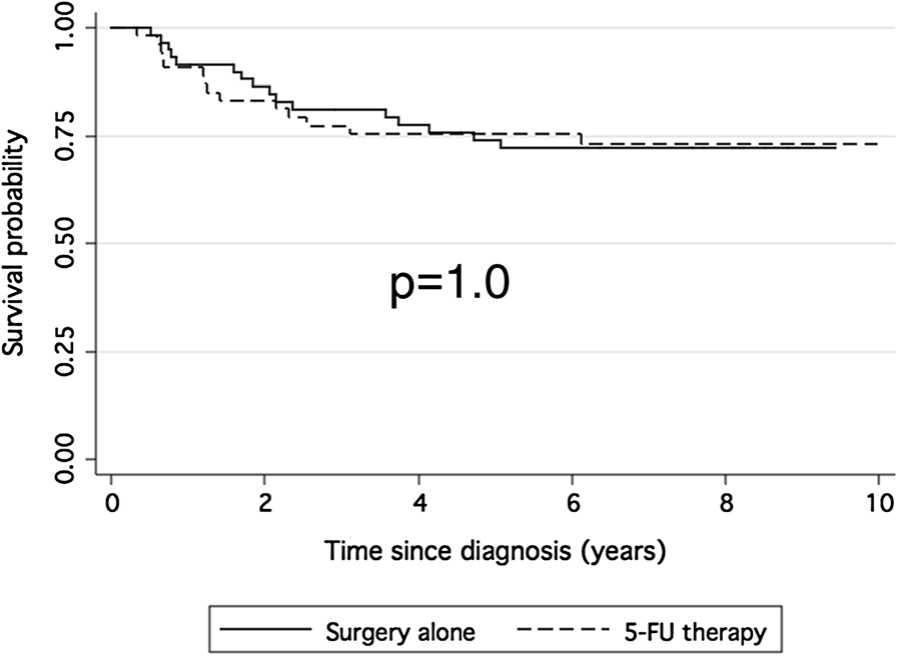
**Survival of the study population as a whole.** Kaplan-Meier curves showing disease free survival in patients treated with surgery alone or with surgery plus adjuvant 5-FU based therapy.

At the end of the follow-up 26 patients died for colon cancer progression, with a median overall survival of 3.2 years (25^th^-75^th^ percentile = 1.9-6.8 yrs) for patients who only received surgical therapy and 2.9 years (25^th^-75^th^ percentile =2.1-3.3 yrs) for those treated with 5-FU. In detail, specific cancer death was recorded for 14 patients who did not receive adjuvant treatment (23%) and for 12 patients (20%) who were treated with adjuvant chemotherapy. The median DFS was of 1.9 years (25^th^-75^th^ percentile = 0.8-3.7 yrs) for the 14 patients who did not receive adjuvant treatment and 1.5 years (25^th^-75^th^ percentile = 0.7-2.3 yrs) for the others. Considering the entire case study, no benefit from adjuvant treatment was detected (Figure 1).

Cox proportional hazard model, including as covariates all clinical, pathological and molecular variables and 5-FU treatment identified BRAF c.1799 T > A mutation as the most important predictor of survival (Table 3). This means that the presence of the mutation c.1799 T > A in the BRAF gene is a poor prognostic factor, independently of 5-FU treatment. The presence of CD8+ lymphocytes infiltrating the tumour, the presence of a lymphocytic reaction at the margin of the tumour as well as the presence of a tumour without an infiltrating border seem to be good prognostic factors, but these data do not reach statistical significance (Table 3).

**Table 3:** **Results of Cox multivariate analysis in the entire patients’ cohort**^**1**^

		**Disease free survival**		**Overall survival**	
		**HR**	**(p)**	**HR**	**(p)**
**Clinical-pathological features**	**Age**	1.93	(0.2)	1.71	(0.3)
	**Sex**	0.70	(0.5)	0.81	(0.7)
	**Tumour location**	1.50	(0.4)	1.85	(0.2)
	**Tumour grade**	1.29	(0.7)	0.81	(0.7)
	**5-FU treatment**	1.23	(0.7)	1.12	(0.8)
	**Depth of tumour invasion**	0.94	(0.9)	1.27	(0.7)
	**Lymphovascular invasion**	0.70	(0.4)	0.61	(0.3)
	**Large veins invasion**	1.08	(0.9)	0.88	(0.8)
	**Tumour inflammatory response**	**0.39**	**(0.06)**	0.40	(0.09)
	**Tumour border configuration**	**2.77**	**(0.06)**	2.91	(0.1**)**
**Molecular parameters**	**MMR system**	1.54	(0.6)	0.97	(1.0)
	**CIMP**	0.58	(0.2)	0.60	(0.3)
	**TS by mRNA**	1.15	(0.7)	1.31	(0.5)
	**TS by IHC**	0.88	(0.8)	0.64	(0.4)
	**TP53**	0.62	(0.2)	0.37	(0.08)
	**CTNNB1activation grades**	0.80	(0.4)	0.90	(0.8)
	**BRAF c.1799 T > A**	**2.57**	**(0.04)**	**3.68**	**(<0.01)**

^1^Significant or borderline p values are bolded.

The cohort of patients was then divided into two groups with respect to the adjuvant treatment, and the Cox proportional hazard regression analysis was repeated separately for the two groups (Table 4). For those patients treated with surgery alone, an independent influence on cancer-progression was detected for the TYMS mRNA expression (p = 0.04; Table 4), while for those patients submitted to adjuvant 5-FU treatment after surgery, the MMR and the CIMP were the most important predictors of survival (p = 0.04 and p = 0.05 respectively; Table 4). Similar results were obtained for the overall survival analysis.

**Table 4:** **Cox multivariate analysis separately shown for patients belonging to the two groups of treatment**^**1**^

		**Surgery alone**		**5-Fu therapy**	
		**HR**	**(p)**	**HR**	**(p)**
**Clinical-pathological features**	**Age**	0.66	(0.6)	3.43	(0.2)
	**Sex**	0.99	(1.0)	1.34	(0.8)
	**Tumour location**	2.09	(0.5)	1.36	(0.7)
	**Tumour grade**	0.71	(0.7)	1.99	(0.7)
	**Depth of tumour invasion**	5.22	(0.2)	1.26	(0.9)
	**Lymphovascular invasion**	0.50	(0.4)	0.27	(0.2)
	**Large veins invasion**	2.14	(0.4)	0.40	(0.3)
	**Tumour inflammatory response**	1.30	(0.6)	0.53	(0.1)
	**Tumour border configuration**	4.91	(0.2)	2.18	(0.6)
**Molecular parameters**	**MMR system**	0.15	(0.2)	**37.85**	**(0.04)**
	**CIMP**	0.67	(0.6)	**0.17**	**(0.05)**
	**TS by mRNA**	**6.08**	**(0.04)**	0.54	(0.2)
	**TS by IHC**	0.35	(0.1)	2.03	(0.4)
	**TP53**	0.71	(0.7)	0.63	(0.6)
	**CTNNB1activation grades**	0.35	(0.1)	1.60	(0.5)
	**BRAF c.1799 T > A**	2.67	(0.3)	2.99	(0.3)

^1^Results of Cox multivariate analysis here reported are referred to DFS (disease free survival). Significant or borderline p values are bolded.

**Figure 2:**
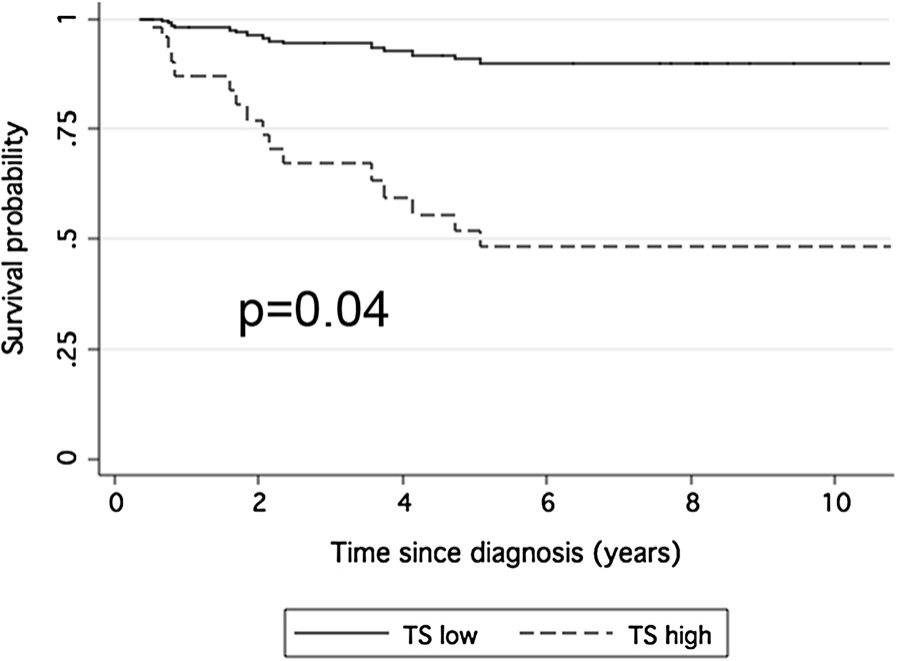
**Disease free survival by TYMS mRNA expression in patients treated with surgery alone.** Survival curves related to Cox proportional hazard regression with respect to TYMS mRNA expression levels in the subgroups of low TYMS (TYMS expression lower than the median value) or high TYMS (TYMS expression higher than the median value).

**Figure 3:**
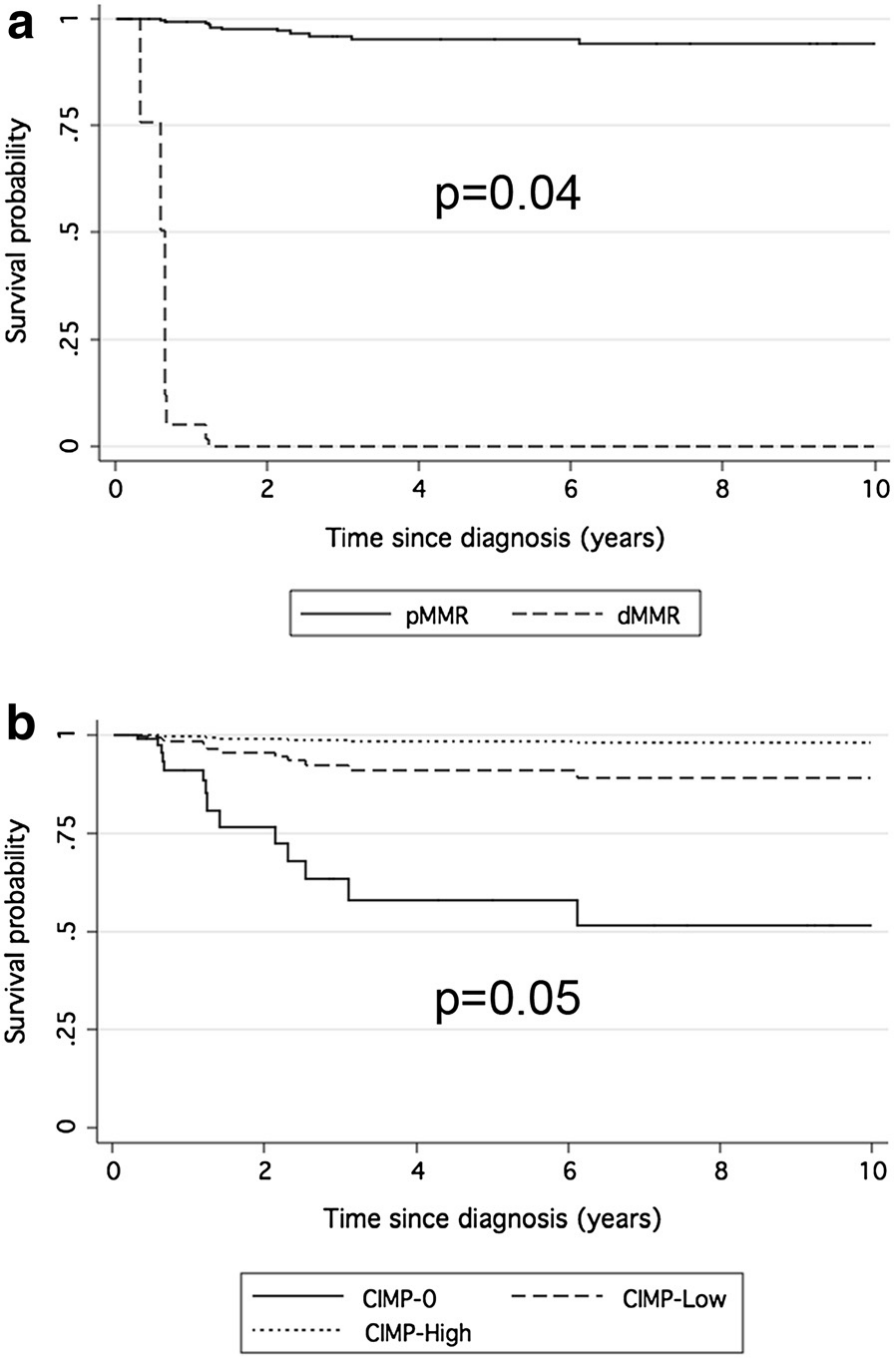
**Disease free survival by MMR and CIMP in patients treated with 5-FU.** Survival curves related to Cox proportional hazard regression with respect to DNA mismatch repair (MMR) status in **a)** and CpG island methylator phenotype (CIMP) in **b)**. dMMR, deficient DNA mismatch repair; pMMR, proficient DNA mismatch repair; CIMP-High, presence of CpG island methylator phenotype; CIMP-Low, presence of low levels of methylation; CIMP-0, absence of CpG island methylator phenotype.

In detail, a low TYMS expression seems to have a protective effect in chemotherapy untreated patients, while in the group of patients who received adjuvant chemotherapy after surgery, the presence of a defect in the MMR system (dMMR) appears pejorative for patients’ survival. In treated patients, conversely, the presence of the CpG island methylator phenotype (CIMP-High) was a protective factor (Figures 2 and 3).

**Figure 4:**
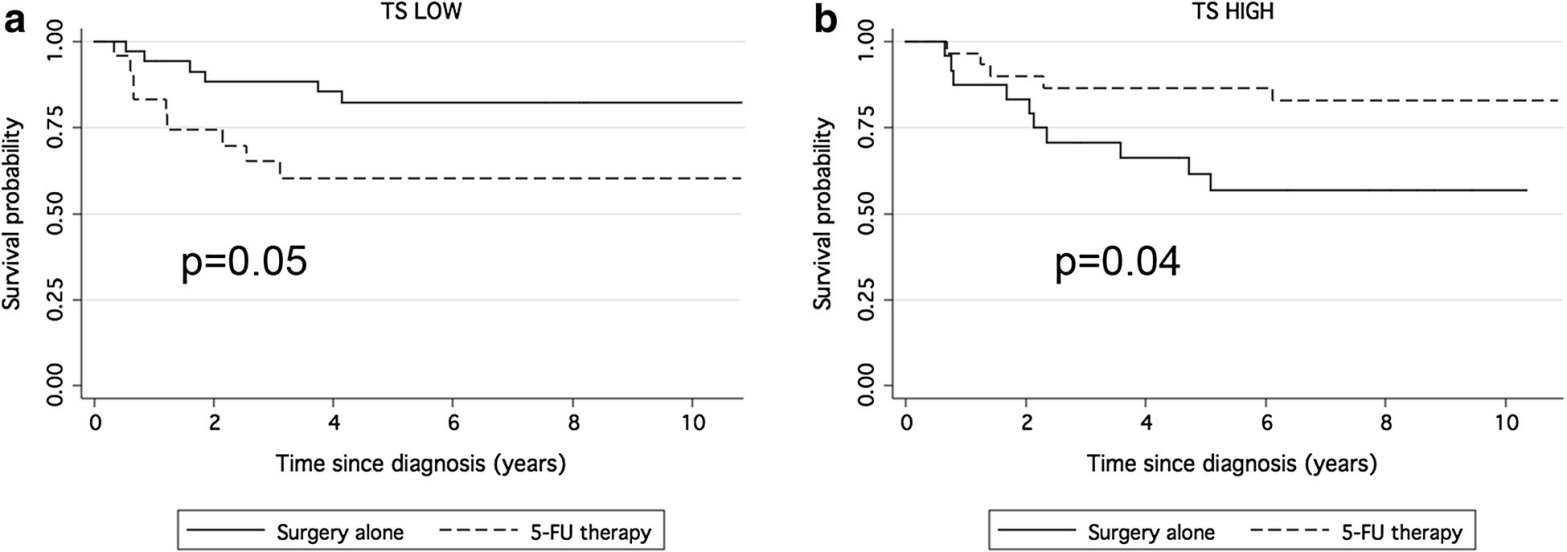
**Disease free survival by 5-FU treatment according to TYMS expression.** Kaplan-Meier survival curves showing disease free survival in patients with low-TYMS-expressing tumours in **a)** and in patients with high-TYMS-expressing tumours in **b)**, with respect to treatment with surgery alone versus surgery followed by adjuvant 5-FU-based chemotherapy.

**Figure 5:**
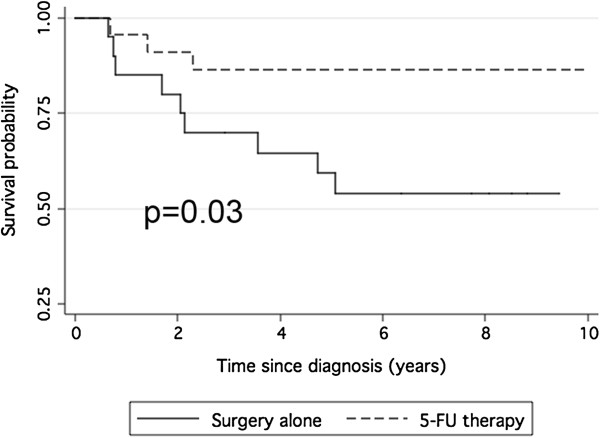
**Disease free survival by 5-FU treatment in pMMR and high TYMS.** Kaplan-Meier survival curves showing disease free survival in patients with pMMR and high-TYMS-expressing tumours with respect to treatment with surgery alone versus surgery followed by adjuvant 5-FU-based chemotherapy.

TYMS mRNA levels, MMR and CIMP statuses have been found as the only markers associated with patients’ survival after their stratification according to treatment, so we hypothesized that these markers could have a predictive role on treatment efficacy. A harmful effect from 5-FU treatment was observed in patients with low TYMS levels (p = 0.05; Figure 4), whereas in patients with high TYMS levels a benefit from 5-FU treatment was found (p = 0.04; Figure 4). No effect from 5-FU therapy was found for patients CIMP-0, CIMP-Low or for those MMR proficient (p = 0.7, p = 0.9 and p = 0.7 respectively). In the latter group, however, high TYMS expression was linked to better outcome after therapy (p = 0.03; Figure 5). These data were also consistent with OS analysis. Of note, in the group of patients showing a low TYMS level, at five years of follow up, overall survival was 91% for patients being treated with surgery alone versus 67% of those treated with 5-FU. The opposite result was seen for patients with high TYMS levels: at five years of follow up, overall survival was 70% for patients being treated with surgery alone versus 90% of those also treated with 5-FU.

Evaluation of 5-FU therapy efficacy in dMMR and CIMP-High tumours was not assessed because of the low number of recurrent patients in these groups (4 cases only for both markers).

## Discussion

Adjuvant therapy in patients with stage II colon cancer is a subject of controversy because of the small gains in survival for this group of patients [7]. Apparently, that was confirmed by our results, showing that the addition of adjuvant treatment to patients with node-negative cancers did not improve DFS and OS (Figure 1). Uncertainty on the benefit of adjuvant chemotherapy in patients with stage II colon cancers is due to the fact that we don’t know which of the stage II patients are at high risk of recurrence and which may benefit from chemotherapy [16]*.* To deepen investigation on these aspects, in the current study a combination of pathological factors and molecular markers was assessed in 120 cases of stage II colon cancer independently of 5-FU treatment.

Considering the entire case study, we found that patients showing c.1799 T > A mutation in the BRAF gene showed a worse prognosis, independently of 5-FU treatment, as already reported [42,43]. In addition, even though non statistically significant, patients having tumours with a pushing border, or showing a tumour inflammatory response, were associated with better survival. The first finding could be explained by the fact that an infiltrating tumour margin is strongly associated with the presence of tumour budding [44], which has been shown to have independent negative prognostic value in colorectal cancer [14]. The latter is related to the reported good prognostic value of generalized inflammatory cell infiltrate in colorectal cancers of stage II patients [45,46] and may be explained by a tumour-related immune response.

Interestingly the adverse effect of BRAF mutation on patients’ outcome appeared to be independent from CIMP phenotype, since in our case study we could not find an association between these two parameters, contrarily to other reports [2,3].

After splitting the group of patients according to treatment, we found a differential effect of 5-FU-based adjuvant therapy according to thymidylate synthetase (TYMS) mRNA levels, the presence of a defect in the MMR system (dMMR) and the presence of the CpG island methylator phenotype, suggesting that these are important parameters for therapy decision. In patients treated with surgery alone, a high TYMS expression was inversely correlated with survival, as previously reported [24,47]. On the contrary, an improved clinical outcome was seen for patients submitted to 5-FU therapy and having a high TYMS expression. Controversial results have been reported on the relationship between TS and 5-FU treatment [24-27,31]. Such contradictory results may be related to differences in patient cohorts and methodological inconsistencies. In particular, our results showed that TYMS levels measured by real time qRT-PCR did not correlate with the corresponding protein levels measured by IHC and the effect on the outcome was evident for TYMS only when evaluated at the mRNA level.

Consistent with other studies [19,48], our data suggest a favourable prognosis in patients with dMMR tumours if untreated with adjuvant therapy (HR, 0.15; p = 0.2) and agree with other reports, indicating that 5-FU treatment should be avoided in patients with dMMR tumours [18,19]. Of note, in our case study, 75% of dMMR patients who developed a recurrence were treated with 5-FU adjuvant therapy. A possible antitumor immune response by the lymphocytic infiltrate characteristic of dMMR tumours [3,19], which may be abrogated by the immunosuppressive effects of chemotherapy, could explain these findings. The favourable prognosis of patients with dMMR cancers and their lack of benefit from 5-FU therapy supports a non-adjuvant treatment approach in these patients, whereas pMMR could be considered as a risk factor to recommend adjuvant chemotherapy [19]. Cancers with a proficient MMR system, however, comprise the majority (80% to 85%) of colorectal cancers and adjuvant therapy decision in such patients should be addressed to other factors [19], for instance TYMS expression, which we found associated to therapy benefit in patients with pMMR tumours (Figure 5).

Different studies reported that CIMP tumours have a distinct clinical, pathological, and molecular profile [3]. Our results confirm the associations between CIMP and proximal location, poor tumour differentiation and MMR deficiency, also showing the recently reported association between CIMP-low and male sex [5]. On the other hand, studies on CIMP phenotype and survival in colon cancer have yielded somewhat contrasting results [21,22,49]. Our findings of good prognosis and therapy benefit in CIMP-High tumours are in agreement with other reports [22,49], even though other studies suggested a lack of benefit from 5-FU adjuvant therapy in these patients [21]. Evidence was also supplied about an interplay between CIMP and MMR status in affecting prognosis [50,51]. Unfortunately the absolute number of dMMR and CIMP-High occurrences are modest in our case study, therefore conclusive results cannot be drawn for the latter consideration. The comparison between our results and the other studies is also complicated by the fact that some studies considered only stage III patients [49], while in others the impact of adjuvant 5-FU chemotherapy relative to the CIMP status was not addressed in specific subgroups (stage II versus stage III patients) [21]. A possible explanation for the apparent chemosensitivity of CIMP-High tumours could be related to the link between CIMP-High and intracellular folate methabolism. It has been reported that CIMP-High tumours show elevated concentrations of intracellular folates, which have a critical importance in determining the response to 5-FU [52]. Moreover, results from microarray analysis have revealed that several genes involved in the pathways of nucleotide metabolism, as well as folate and glutamine metabolism are differentially expressed between CIMP-positive and CIMP-negative tumours [53]. Of note, the reported study also shows that TYMS levels are significantly higher in CIMP-positive tumours with respect to CIMP-negative ones, confirming our results [53]. We also found higher TYMS levels in patients with a defect in the MMR system, in agreement with Ricciardiello et al. [54]. We recognise that the relationship between TYMS, MMR, and CIMP in colon cancer could be complex. In our cohort, 50% of CIMP-High tumours showed a defect in the MMR system and 72% of dMMR tumours exhibited a high TYMS level. High TYMS levels were more common also in patients with the CpG island methylator phenotype, but while TYMS expression and CIMP-High status have been associated to a positive outcome in patients treated with 5-FU, dMMR has been linked to worse outcome, highlighting the complexity of these molecular relationships. Stratification of patients according to combinations of TYMS expression, MMR and CIMP statuses to assess their joint effect on patient outcome was not performed in this study because of the small number of patients. Larger case-studies will be necessary to deepen investigation on the prognostic and predictive role of such markers in patients with stage II colon cancers, also adopting a multisegmental approach to bowel subsites [55] in order to improve tailored preventive and therapeutic strategies.

## Conclusions

To conclude, this study supports the contention that TYMS expression (at the mRNA level), dMMR and CIMP-High status are clinically relevant markers in patients with sporadic stage II colon cancer being considered for fluoropyrimidine-based adjuvant therapy. In particular, this study suggests that patients with high TYMS levels and CIMP-High status may derive benefit from adjuvant therapy, which however should be avoided in those showing dMMR and low TYMS tumours. Nevertheless the limited sample size of this study prevents definite conclusions. Further studies with higher power in sample sizes will be needed to deeply investigate the roles of TYMS expression, dMMR and CIMP status in patients with stage II colon cancer.

## Abbreviations

5-FU: Five-Fluorouracil;CIMP: CpG island methylator phenotype;DFS: Disease free survival;FFPE: Formalin-fixed paraffin embedded;MMR: Mismatch repair system;MSI: Microsatellite instability;OS: Overall survival;TYMS: Thymidylate synthetase

## Competing interests

The authors declare that they have no competing interests.

## Authors’ contributions

MD carried out all the molecular analyses and contributed in analysing data and writing the manuscript. SB performed the statistical analyses, contributed in writing the manuscript and critically revised it. RB performed the histological revision of the case study and evaluated the immunohistochemistry (IHC). DP contributed in CIMP and BRAF molecular analyses. GS had the intellectual property of the study designing it and contributing in revising it in all steps. All authors read and approved the final manuscript.

## Pre-publication history

The pre-publication history for this paper can be accessed here:

http://www.biomedcentral.com/1471-230X/13/36/prepub
